# Supplementary material for: Anti-Glycolipid Antibody Examination in Five EAE Models and Theiler’s Virus Model of Multiple Sclerosis: Detection of Anti-GM1, GM3, GM4, and Sulfatide Antibodies in Relapsing-Remitting EAE
Source: Int J Mol Sci. 2023 Aug 18;24(16):12937. doi: 10.3390/ijms241612937 (PMC10454742; doi:10.3390/ijms241612937)
Supplement: Supplementary file 1 [file ijms-24-12937-s001.zip › ijms-2558044-supplementary.pdf]

Supplementary Table S1. Correlations of anti-glycolipid antibody levels with EAE scores in PLP-sensitized mice[a]

Anti-glycolipid antibody	$PLP_{139–152}$-sensitized mice (n = 18)[b]			symptomatic EAE mice (n = 17)[c]			EAE mice with limb paralysis (n = 15)[d]		
	r_s	p	Power[e]	r_s	p	Power	r_s	p	Power
GM1	−0.34	0.16	0.29	**−0.51**	**< 0.05**	0.58	**−0.74**	**< 0.01**	0.92
GM3	−0.17	0.49	0.10	−0.24	0.34	0.16	−0.42	0.12	0.36
GM4	0.01	0.98	0.05	−0.23	0.33	0.15	**−0.60**	**< 0.05**	0.70
sulfatide	−0.11	0.66	0.07	−0.16	0.55	0.10	−0.23	0.39	0.13

[a]We sensitized 18 SJL/J mice with $PLP_{139–151}$, collected sera from the mice on days 14–21, and determined serum anti-glycolipid antibody levels ($Abs_{492\ nm}$) by ELISA. Neurological signs were evaluated using EAE scores. Since both $Abs_{492\ nm}$ and EAE scores are non-parametric data, we used Spearman rank correlation to analyze the association between the antibody levels and EAE scores [66].

[b]In most EAE models, the induction rate of full-blown EAE (with hind limb paralysis, EAE scores ≥2) was not 100%. In this study, we found 15 of 18 (83%) mice had EAE scores of 2 or higher on days 14–21; one mouse did not develop any EAE signs, and two mice had only tail paralysis. We analyzed the correlations using data from all 18 mice sensitized with $PLP_{139–151}$ and found no significant correlation ($P > 0.05$).

[c]When we used the data from all symptomatic EAE mice, excluding one asymptomatic mouse (EAE score = 0), anti-GM1 antibody levels had a moderate negative correlation with EAE scores statistically ($r_s = −0.51$, $p < 0.05$). We interpreted a Spearman correlation coefficient ("r_s" or ρ) based on Mukaka (2012) as follows: 0.7 to 1.0 (−0.7 to −1.0) high positive (negative) correlation; 0.5 to 0.7 (−0.5 to −0.7), moderate positive (negative) correlation; 0.3 to 0.5 (−0.3 to −0.5), low positive (negative) correlation; and 0 to 0.3 (0 to −0.3) negligible correlation.

[d]When we used the data from mice who developed hind limb paralysis (EAE score = 2 or higher), excluding one asymptotic mouse and two mice who had only tail paralysis (EAE score = 1), anti-GM1 antibody levels had a high negative correlation with EAE scores ($r_s = −0.74$, $p < 0.01$); anti-GM4 antibody titers had a moderate negative correlation with EAE scores statistically ($r_s = −0.6$, $p < 0.05$).

[e]Power analysis was conducted, using an R version 4.3.0 and the package "WebPower" version 0.9.3 [67,68].
